# Deep Learning-Based Universal Expert-Level Recognizing Pathological Images of Hepatocellular Carcinoma and Beyond

**DOI:** 10.3389/fmed.2022.853261

**Published:** 2022-04-22

**Authors:** Wei-Ming Chen, Min Fu, Cheng-Ju Zhang, Qing-Qing Xing, Fei Zhou, Meng-Jie Lin, Xuan Dong, Jiaofeng Huang, Su Lin, Mei-Zhu Hong, Qi-Zhong Zheng, Jin-Shui Pan

**Affiliations:** ^1^Liver Research Center, The First Affiliated Hospital of Fujian Medical University, Fuzhou, China; ^2^School of Medicine, Xiamen University, Xiamen, China; ^3^School of Aerospace Engineering, Xiamen University, Xiamen, China; ^4^Department of Anesthesiology, Zhongshan Hospital Xiamen University, Xiamen, China; ^5^Department of Gastroenterology, Zhongshan Hospital Xiamen University, Xiamen, China; ^6^Department of Pathology, Zhongshan Hospital Xiamen University, Xiamen, China; ^7^Department of Traditional Chinese Medicine, Mengchao Hepatobiliary Hospital of Fujian Medical University, Fuzhou, China; ^8^Department of Pathology, Xiamen Hospital of Traditional Chinese Medicine, Xiamen, China

**Keywords:** machine learning, pathology, transfer learning, diagnostic imaging, hepatocellular carcinoma

## Abstract

**Background and Aims:**

We aim to develop a diagnostic tool for pathological-image classification using transfer learning that can be applied to diverse tumor types.

**Methods:**

Microscopic images of liver tissue with and without hepatocellular carcinoma (HCC) were used to train and validate the classification framework based on a convolutional neural network. To evaluate the universal classification performance of the artificial intelligence (AI) framework, histological images from colorectal tissue and the breast were collected. Images for the training and validation sets were obtained from the Xiamen Hospital of Traditional Chinese Medicine, and those for the test set were collected from Zhongshan Hospital Xiamen University. The accuracy, sensitivity, and specificity values for the proposed framework were reported and compared with those of human image interpretation.

**Results:**

In the human–machine comparisons, the sensitivity, and specificity for the AI algorithm were 98.0, and 99.0%, whereas for the human experts, the sensitivity ranged between 86.0 and 97.0%, while the specificity ranged between 91.0 and 100%. Based on transfer learning, the accuracies of the AI framework in classifying colorectal carcinoma and breast invasive ductal carcinoma were 96.8 and 96.0%, respectively.

**Conclusion:**

The performance of the proposed AI framework in classifying histological images with HCC was comparable to the classification performance achieved by human experts, indicating that extending the proposed AI’s application to diagnoses and treatment recommendations is a promising area for future investigation.

## Introduction

Hepatocellular carcinoma (HCC) is the fifth most common cancer worldwide and the second most common cause of cancer-related deaths (1). In the United States and China, HCC is estimated to be the fourth and third most common cause of cancer-related deaths, respectively (2, 3). This liver cancer develops in patients with liver cirrhosis, especially in patients with chronic hepatitis B (CHB) or chronic hepatitis C (CHC)-related liver cirrhosis (4–6). In cirrhosis cases, several nodules of varying sizes are found in the liver. As they are highly similar, identification of benign and malignant intrahepatic nodule is often considerably challenging for computed tomography (CT) or magnetic resonance imaging (MRI)-based diagnosis; in selected cases, for example, in case of healthy liver or atypical imaging presentation, the definitive diagnosis depends on liver biopsy. Histopathology is the gold standard for determining the nature of hepatic space occupying lesions; however, diagnosing a large number of pathology slide images is laborious, and the substantial observer-to-observer variation in liver biopsy assessments cannot be neglected (7). Another challenge in medical-image diagnostics is patient-to-patient variability in the pathology of disease manifestation. Even experienced pathologists provide significantly different interpretations regarding the histopathology of the same disease. Therefore, novel auxiliary diagnostic facilities should be developed.

Diagnostic approaches to HCC include ultrasound, CT, and MRI (8). In addition to HCC, colorectal cancer (CRC) and breast invasive ductal carcinoma (BIDC) are some of the most common tumors. In 2015, CRC was estimated to be the fifth most common cause of cancer-related deaths in China (3). Similarly, according to the 2018 United States cancer statistics published by Siegel et al. (2). CRC is the third most common cause of cancer-related deaths in both men and women. Moreover, adenocarcinoma is the most common type of CRC whose diagnosis depends on pathology imaging and interpretation. Diagnostic methods for CRC include CT, colonoscopy, and subsequent tissue examination (9), whereas breast cancer diagnostics include ultrasound and mammography (10). In 2015, breast cancer was estimated to have contributed toward most new cancer cases in China (3). The same was true in the United States, according to cancer statistics published by Siegel et al. (2). Invasive ductal carcinoma is the most common type of breast cancer diagnosed histologically (11). Similar to CRC, histopathology examination is the diagnostic gold standard for BIDC. In summary, several imaging approaches in diagnostics for almost all diseases are available; further, almost all diagnostic imaging approaches produce a large number of medical images. For example, a plain scan combined with contrast-enhanced CT or MRI can produce more than 1,000 images per examination, whereas capsule endoscopy can produce more than 40,000 medical images per examination. Further, the interpretation of these images can be time consuming.

The number and types of medical images have expanded at an unprecedented rate owing to the continuous emergence of new technologies. However, the challenge of handling the imbalance between the ability and number of specialized practitioners and the expanding medical imaging output remains unsolved. A physician may be familiar with only a few or even just one type of diagnostic imaging technique, whereas the interpretation of countless medical images requires human expertise and judgment to correctly understand and triage. Therefore, an artificial intelligence (AI) system that can achieve high classification accuracy with a universal recognition capability should be developed.

In recent years, AI has been widely used in various fields (12–17). Several studies have reported the application of AI in the diagnosis of HCC. With the assistance of AI, the accuracy of diagnosis of HCC is significantly improved (18–20). More than that, the deep learning framework is helpful for accurate HCC segmentation from whole-slide images (21, 22). In addition, machine learning offers potential as an effective and labor-saving method for postoperative follow-up observation and HCC risk stratification (23, 24). For classification tasks that are difficult for human experts or where the rapid review of a large number of images is required, AI has outstanding advantages, such as savings in time, high accuracy, and low volatility. AI plays a revolutionary role in disease diagnosis. In this study, we develop an effective convolutional neural network (CNN) based on a deep-learning algorithm to classify medical images. Then, we evaluate the generalizability performance of the proposed AI system in interpreting histological images of several common types of tumors through transfer learning.

## Materials and Methods

### Patients

Patients who underwent biopsy or surgical resection because of the diseases of the liver, colorectum, or breast in the Xiamen Hospital of Traditional Chinese Medicine, or the Zhongshan Hospital Xiamen University between June 1, 2010, and December 31, 2017, were selected. Among these, adult patients aged between 18 and 75 were enrolled in the study. The inclusion criteria included biopsy or surgical resection specimens with a completed structure, and one of the following conditions: (1) chronic hepatitis-related to hepatis B virus (HBV) or hepatis C virus (HCV), without HCC, with or without liver cirrhosis; (2) HCC companied by HBV-related or HCV-related chronic hepatitis, with or without liver cirrhosis; (3) CRC; and (4) BIDC.

All HCC, CRC, and BIDC were further confirmed based on the surgically resected specimens. Necroinflammatory activity and fibrosis or cirrhosis related to chronic hepatitis were recorded using the Scheuer system (25). The histological diagnosis of HCC or CRC was performed using the digestive tumor-classification system formulated by the World Health Organization (WHO) in 2010 (26), while the histological diagnosis of BIDC was performed using the breast-tumor-classification system formulated by WHO in 2012 (27). No exclusion criteria regarding gender or race exist. This study was approved by the Ethics Committees of First Affiliated Hospital of Fujian Medical University. Written informed consent was waived by the Ethics Commission of the designated hospital because of the non-interventional nature of the study and because no identifiable personal information was recorded. All experiments were performed in accordance with the relevant guidelines and regulations.

### Images

The collected tissue samples were fixed in wax, followed by slicing to a thickness of 3 μm. Then, the samples were stained using hematoxylin-eosin. After staining, histological images were collected with a 200-fold magnification. A total of 2–6 images were collected for each patient, and no overlap was observed between the images. For a case with a tumor, an image of the tumor was collected accompanied by a corresponding non-tumor image, which was captured 2 cm away from the tumor. The strategy of the “full field” was adopted. An image of the tumor was captured near the tumor, whereas an image of a non-tumor was captured away from the tumor. In other words, the entire image of the tumor comprised tumor tissue, while the entire non-tumor image comprised non-tumor tissue. Each image was examined by a panel of two independent pathologists, each with over 15 years of pathology experience. If a disagreement in clinical labeling exists, the image was further arbitrated by a panel of senior pathology specialists. Initially, the size of the pathological images collected using the optical microscope was 1,920 × 1,280. We resized these images into 224 × 224 pixels before being sent to the CNN for training. Identifiable personal information, such as name of the enrolled patients and name of hospital, was removed.

### Datasets

Histological images collected from the Department of Pathology, Xiamen Hospital of Traditional Chinese Medicine, were used as training and validation sets, while those collected from the Department of Pathology, Zhongshan Hospital Xiamen University, were used as test sets to further verify the classification performance. Histological images of HCC, non-HCC, CRC, non-CRC, BIDC, and non-BIDC were collected independently from these two hospitals in the same manner.

### Training and Validation of the Artificial Intelligence Algorithm

Each divided image of 1,920 × 1,280 pixels was imported into the database with multiple layers of classification. An entire image was classified as a “tumor” if a tumor was identified even in one dissected image. However, the image was classified as “non-tumor” only when all dissected images were recognized as “non-tumor.” Collected liver pathology images were randomly divided into training and validation sets in a ratio of 3:1. The training set was used to train the AI algorithm whereas the validation set was employed to evaluate the classification performance of the trained AI algorithm. This process was repeated five times.

Based on deep learning, we developed an AI algorithm and used the PyTorch platform to adopt the ResNet-34 architecture pretrained using the ImageNet dataset (28). Retraining comprised the initialization of the convolutional layers with loaded pretrained weights and update of the neural network to recognize our classes, such as HCC and non-HCC. The network structure remained unchanged during the training process. However, in addition to the last fully connected layer, the network learning rates were tuned to 0.001. The learning rate of the last fully connected layer was tuned to 0.02 (0.001 × 20), and the weights were updated using backpropagation. This strategy tended to update the first several layers slowly while updating the output layer more efficiently. Layer training was performed by stochastic gradient descent in batches of 64 images per step using a stochastic gradient descent optimizer. The training procedure was run for 25 epochs with a dropout ratio equal to 0.5. The modified ResNet-34 was trained on a 14.04.1 Ubuntu computer with Intel (R) i7-5930K CPU @ 3.50 GHz. An NVIDIA GTX 1080Ti 11 GB GPU was utilized to accelerate training.

### Testing the Artificial Intelligence Algorithm

After the training process was finished, the histological images collected from the Department of Pathology, Zhongshan Hospital Xiamen University, were used as the test set to monitor the classifying decisions of the trained algorithm.

### Comparison Between the Artificial Intelligence Algorithm and Human Experts

Histological images collected from the Department of Pathology, Zhongshan Hospital, Xiamen University, were also sent to expert pathologists for diagnosis. Their classification performance was compared with that of the AI algorithm. The expert pathologists were part of the senior staff at the Department of Pathology, Zhongshan Hospital, Xiamen University, and they each had a clinical experience of approximately 15 years. The diagnosis was conducted independently; the error rates were determined for the AI algorithm and for each human expert. Further, the performances of the proposed AI algorithm and other frameworks, such as AlexNet and GoogLeNet, were compared (29, 30).

### Transfer Learning of the Artificial Intelligence Algorithm

Transfer learning was developed by Donahue et al. (31). To evaluate the transfer learning performance, the trained AI algorithm was further tested using two other types of tumors: CRC and BIDC. Specifically, the classification performance of the trained AI algorithm was determined independently for each type of tumor.

**FIGURE 1:**
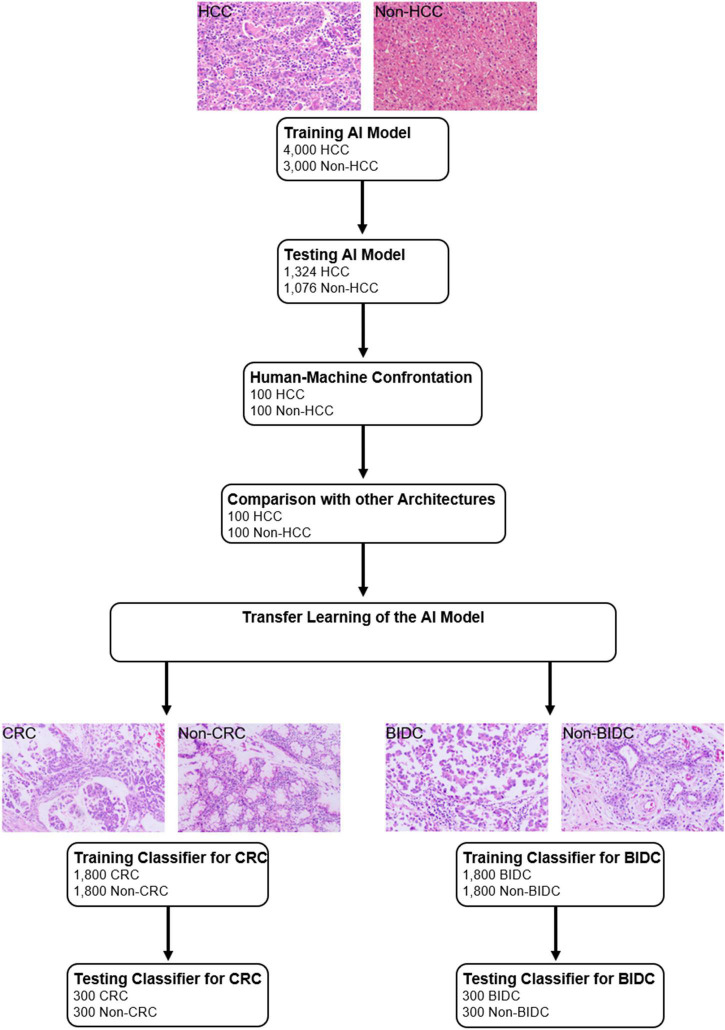
Study design.

The study design is shown in Figure 1, and the CNN schematic for HCC classification is shown in Supplementary Figure 1.

### Statistical Analysis

To evaluate the classification performance of the AI algorithm on histological images, three indexes, namely, accuracy, sensitivity, and specificity, were calculated. The receiver operating characteristics (ROC) curves plot the true-positive rate (sensitivity) vs. the false-positive rate (1-specificity). *P* < 0.05 was set as the level for statistical significance for two-tailed paired test.

### Patient Consent Statement

This study was approved by the Ethics Committees of First Affiliated Hospital of Fujian Medical University.

## Results

### Patient and Image Characteristics

We obtained 7,000 liver pathology slide images generated from 2,745 patients enrolled from the Xiamen Hospital of Traditional Chinese Medicine, where 4,000 images showed confirmed HCC, while the other 3,000 images confirmed other diseases, such as CHB/CHC with or without cirrhosis. All images passed an initial image quality check, and they were randomly divided into training and validation sets at a ratio of 3:1 to train and validate the classification performance of the AI algorithm. This process was repeated five times.

### Artificial Intelligence Algorithm Performance During Training and Validation

During training and validation, the accuracy and cross-entropy were plotted against the iteration step, as shown in Supplementary Figure 2. Using the validation set as the reference, the mean sensitivity, specificity, and accuracy of the AI algorithm were calculated as 98.6, 98.5, and 98.5%, respectively.

### Artificial Intelligence Algorithm Performance Evaluated Using the Test Set

We generated 2,400 images with or without HCC from 873 patients that enrolled from the Zhongshan Hospital, Xiamen University; these images were used to further evaluate the performance of the AI algorithm. In these 2,400 images, 1,324 showed HCC, while 1,076 showed non-HCC. Using the test set as the reference, the sensitivity, specificity, and area under the ROC curve of the AI algorithm were calculated as 99.1, 98.0, and 96.0, respectively.

### Comparison Between the Results of the Artificial Intelligence Algorithm and Human Experts

**FIGURE 2:**
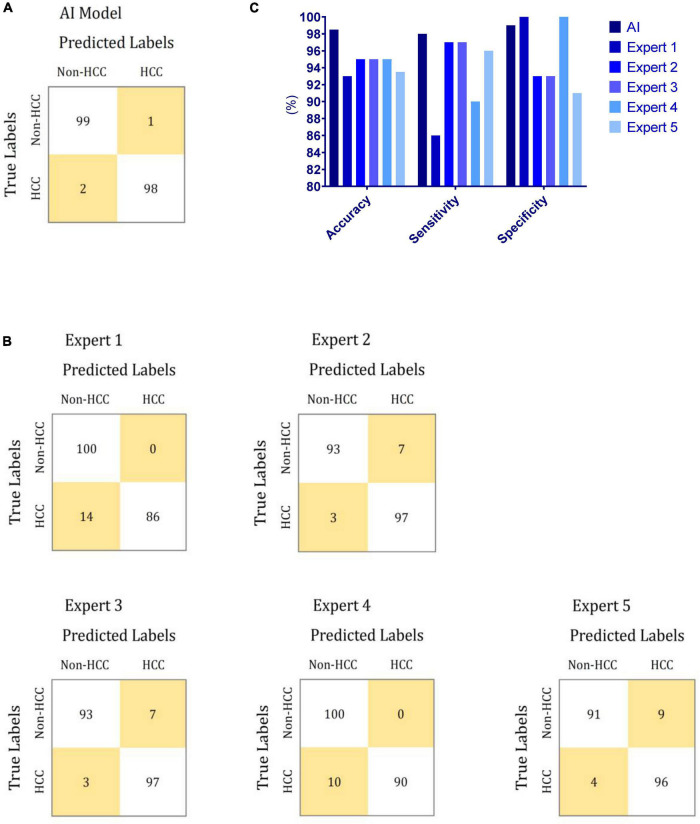
Performances of the proposed AI model and human experts during human–machine comparison. **(A)** Confusion matrix of the proposed AI model for HCC diagnosis. **(B)** Confusion matrix of human experts for HCC diagnosis. **(C)** Comparison between the performances of the proposed AI model and human experts for HCC diagnosis.

To compare the performances of the AI algorithm and human experts, we chose another randomly selected set of 200 images comprising 100 images each with and without HCC. All 200 images were sent to both the AI algorithm and human experts for clinical decisions. The accuracy, sensitivity, and specificity for the AI algorithm were 98.5, 98.0, and 99.0%, respectively. For the human experts, the sensitivity ranged between 86.0 and 97.0%, while the specificity ranged between 91.0 and 100%. Compared with the human experts, the AI algorithm tended toward a more balanced performance between sensitivity and specificity. However, a remarkable variation was observed between sensitivity and specificity, which distinguished the performance of the AI algorithm from that obtained by human experts. The performances of the AI algorithm and human experts are presented in Figure 2.

### Comparison Between the Artificial Intelligence Algorithm and Other Architectures

**FIGURE 3:**
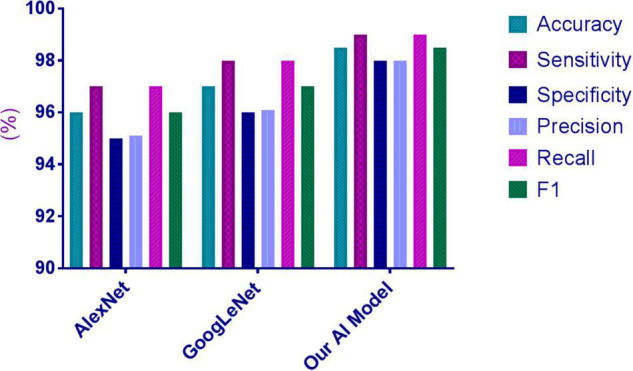
Performances of the proposed AI model and other architectures for HCC diagnosis.

The 200 images used for the human–machine comparison were employed to compare the performance of the AI algorithm with that of the other architectures. The HCC image-recognition sensitivity, specificity, and accuracy of the proposed AI system were superior to those of AlexNet and GoogleNet, as reported in Supplementary Table 1 and Figure 3.

### Transfer Learning of the Artificial Intelligence Algorithm to Colorectal Cancer

**FIGURE 4:**
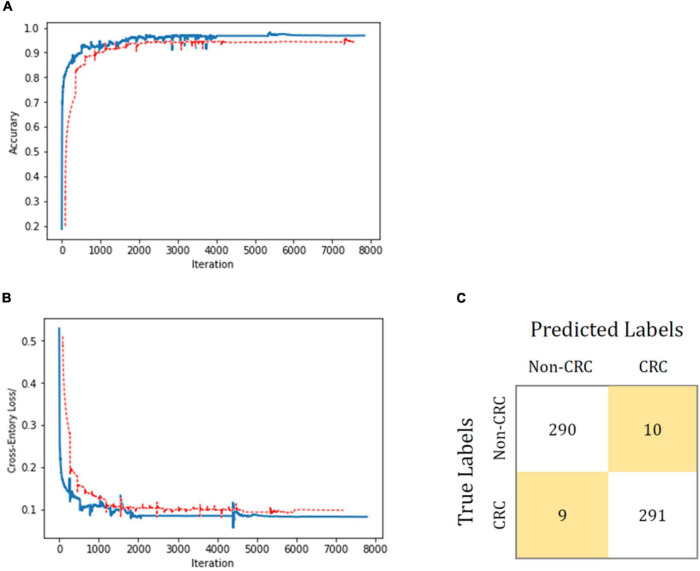
Transfer-learning performance of CRC diagnosis using colorectal tissue microscope slide images. In **(A,B)**, the training dataset is shown in blue, and the test dataset is shown in red. Accuracy is plotted against the iteration step **(A)**, and cross-entropy loss is plotted against the iteration step **(B)** during the length of the training of the binary-class classifier over the course of 8,000 steps. The curve is smoothed; the test accuracy and loss show better performance. **(C)** Shows the confusion matrix of the best test image model classification. The model successfully classifies CRC separately from the non-CRC.

To evaluate the proposed transfer-learning performance of the AI system, 3,600 colorectal-tissue microscope slide images were collected from Xiamen Hospital of Traditional Chinese Medicine to train and validate the AI algorithm. These 3,600 images comprised 1,800 images each with and without CRC. Another 600 colorectal-tissue microscope images obtained from Zhongshan Hospital Xiamen University were used as the test set. As shown in Figure 4 and Supplementary Table 2, after only limited training, the proposed AI algorithm showed excellent accuracy in CRC and non-CRC image classification based on transfer learning. An accuracy of 96.8% was achieved with a sensitivity of 97.0% and a specificity of 96.7%.

### Transfer Learning of the Artificial Intelligence Algorithm to Breast Invasive Ductal Carcinoma

**FIGURE 5:**
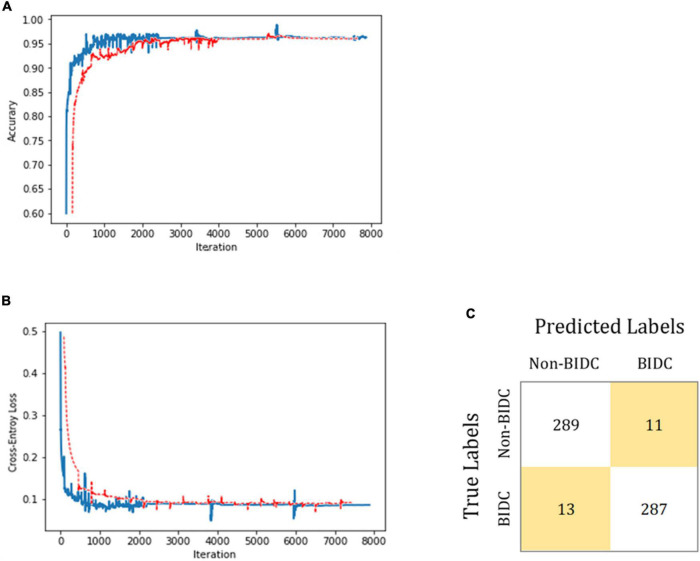
BIDC diagnosis transfer-learning performance using breast tissue microscope slide images. In **(A,B)**, the training and test datasets are shown in blue and red, respectively. The classification accuracy is plotted against training epochs, and in **(B)**, the categorical cross-entropy loss is shown as a function of training epochs for the binary classification problem. The curve is smoothed. **(C)** Shows the model-classification confusion matrix for test image classification. As shown, the proposed model successfully classifies BIDC from non-BIDC images.

Microscope slide images from breast tissue were collected to further evaluate the transfer-learning performance of the proposed AI system. A total of 3,600 histologic images of the breast obtained from Xiamen Hospital of Traditional Chinese Medicine were employed to train and validate the AI algorithm. These 3,600 images comprised 1,800 images each with and without BIDC. Another 600 breast microscope images were obtained from the Zhongshan Hospital Xiamen University as a test set. As shown in Figure 5, after training, the proposed AI system showed an accuracy of 96.0%, with a sensitivity of 95.7% and a specificity of 96.3% in classifying images into BIDC or non-BIDC.

## Discussion

In this paper, we described a general AI algorithm for the interpretation of histological images from the liver, colorectum, and breast. Although medical imaging techniques such as CT and MRI are widely used for HCC diagnosis, CT and MRI detection show a poor performance for HCCs < 1.0 cm, especially for patients with cirrhosis (32). For those patients without definitive findings on either CT or MRI, a biopsy may be the only detection method (1). Owing to potential interobserver bias that may be present when reviewing histological images generated from biopsy, AI may be considered a useful ancillary tool for HCC identification.

Several architectures have been proposed for a classification task. We evaluated many of these architectures, such as ResNet-34, ResNet-50, and DenseNet; however, we did not observe any significant differences between the classification results of these architectures. Instead, we observed that the performance of ResNet-34 was slightly better than other models (Supplementary Tables 3, 4). Thus, we selected ResNet-34 as our baseline architecture.

We used the Tissue Microarray Images dataset to pre-train ResNet-34. We employed data augmentation to enhance the model’s robustness against color. The parameters included brightness, contrast, and saturation, and thresholds of these three parameters were 0.8–1.2, 0.75–1.25, and 0.9–1.1, respectively. Finally, we used our labeled dataset to train the last three convolution layers and the fully connected network of ResNet-34. The learning rate for the three convolutional layers in training was tuned to 0.001, whereas that of the fully connected layer was tuned to 0.02.

Further, the proposed model demonstrated competitive performance for analysis of liver histological images. This was accomplished without the need for a highly specialized deep learning machine and without using a very large training database. When the model was trained with 7,000 images (3,500 images for each class), high performance accuracy, sensitivity, and specificity were achieved for the correct diagnosis. Moreover, the performance of the model in diagnosing HCC was comparable to that of diagnosis by experts with significant clinical experience in liver pathology.

By employing another set of 200 images (100 images for each class) as the test set, the proposed AI model showed a more balanced performance between sensitivity and specificity in recognizing HCC compared with that of human experts. The accuracy of the proposed AI model was superior to that of experts, indicating a remarkable variation between sensitivity and specificity. The abovementioned test set was used for comparison between the proposed ResNet-34-architecture-based AI model and other AI architectures including AlexNet and GoogleNet. The proposed AI model achieved superior accuracy, sensitivity, and specificity, thus demonstrating the robustness of the model.

During model construction, we observed that the last three convolution layers can help improve classification performance. Inherent differences exist in the process of pathological section staining, and therefore, the final rendering effect of the pathological images inevitably has obvious differences. To overcome this deficiency, we employed data augmentation to improve our models, including randomly changing the brightness, contrast, and color saturation of the image.

Kermany et al. (14) employed over 100,000 OCT images to train the AI framework. In comparison, only 3,600 images were used in our study to train our AI system, but an excellent diagnostic performance was achieved. Thus, even with a limited training dataset, the transfer-learning system demonstrated highly effective classifications.

Transfer-learning techniques for image analysis could potentially be employed for a wide range of medical images across multiple disciplines. In fact, a direct illustration of its wide applicability in the analysis of two similar histological image types (CRC and BIDC) was shown. After a considerably smaller amount of training, the proposed AI model reported accuracies of 96.8 and 96.0% for CRC and BIDC, respectively. The proposed AI model showed balance between sensitivity and specificity. Thus, the proposed AI system has potential universality in the classification of histological images.

An AI model trained using an extremely large training dataset would have superior performance to that of a transfer-learning-based model trained from a relatively small training dataset. However, in practice, the *de novo* training of a CNN needs an unlimited supply of training data, and it requires weeks to achieve good accuracy. Using the retraining layers from other medical classifications, a transfer-learning-based model yields a highly accurate model in considerably less time. Thus, for difficult-to-collect medical images, transfer-learning-based image recognition is more practical. Recently, several studies have highlighted the value of transfer learning in medical image recognition (33–35). Given that imaging-based diagnosis played a crucial role in guiding treatment, extending the proposed AI’s application to diagnoses and treatment recommendations is a promising area for future investigation.

### Limitations

This study has several limitations; these are listed below:

1) No further analysis on the learned features was made in the present study.
2) The amount of the employed images was limited and needs to be expanded in the future.
3) This study focuses on classification rather than detection.

Despite these limitations, this study is considered valuable for exploring AI architecture based on transfer learning for the recognition of the diseases that are difficult in collecting enough images for training.

## Data Availability Statement

The original contributions presented in the study are publicly available. This data can be found here: https://github.com/DarcyFu/Liver-Detection/blob/main/train.py.

## Author Contributions

J-SP and Q-ZZ: study concept and design. Q-ZZ, W-MC, C-JZ, Q-QX, FZ, M-JL, XD, JH, SL, and M-ZH: acquisition of data. MF, W-MC, and J-SP: analysis and interpretation of data. J-SP: drafting of the manuscript. J-SP, Q-ZZ, and M-ZH: critical revision of the manuscript for important intellectual content. M-ZH: administrative, technical, material support, and study supervision. All authors contributed to the article and approved the submitted version.

## Conflict of Interest

The authors declare that the research was conducted in the absence of any commercial or financial relationships that could be construed as a potential conflict of interest.

## Publisher’s Note

All claims expressed in this article are solely those of the authors and do not necessarily represent those of their affiliated organizations, or those of the publisher, the editors and the reviewers. Any product that may be evaluated in this article, or claim that may be made by its manufacturer, is not guaranteed or endorsed by the publisher.
