# Supplementary material for: Deep Learning-Based Universal Expert-Level Recognizing Pathological Images of Hepatocellular Carcinoma and Beyond
Source: Front Med (Lausanne). 2022 Apr 22;9:853261. doi: 10.3389/fmed.2022.853261 (PMC9072864; doi:10.3389/fmed.2022.853261)
Supplement: Supplementary file 1 [file Data_Sheet_1.pdf]

Supplementary Material

1. Supplementary Figures

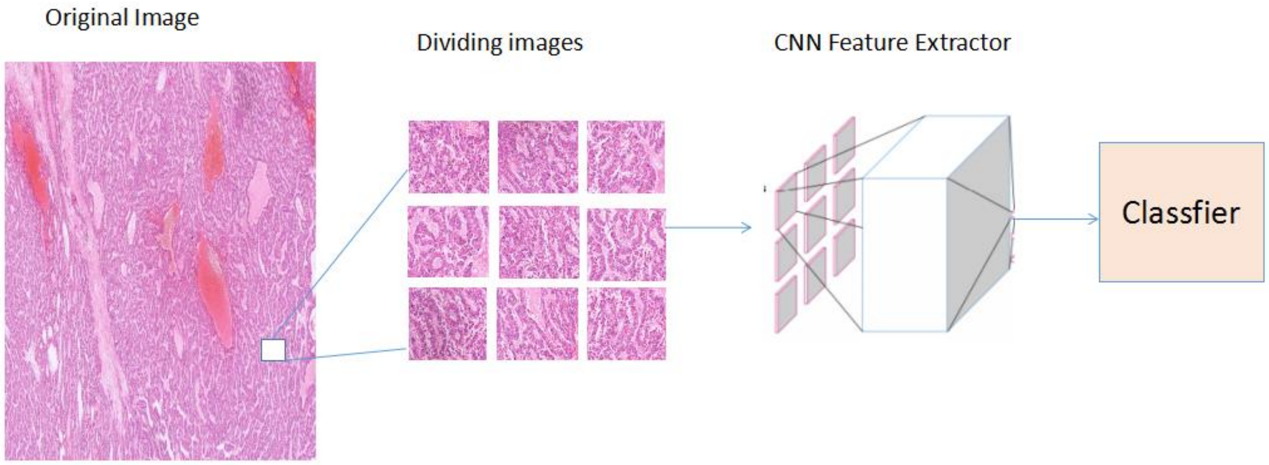

Supplementary Figure 1. Algorithm Architecture. Whole slide images were divided into n images, which were sent to the CNN feature extractor. Finally, the image features were determined by the constructed classifier.

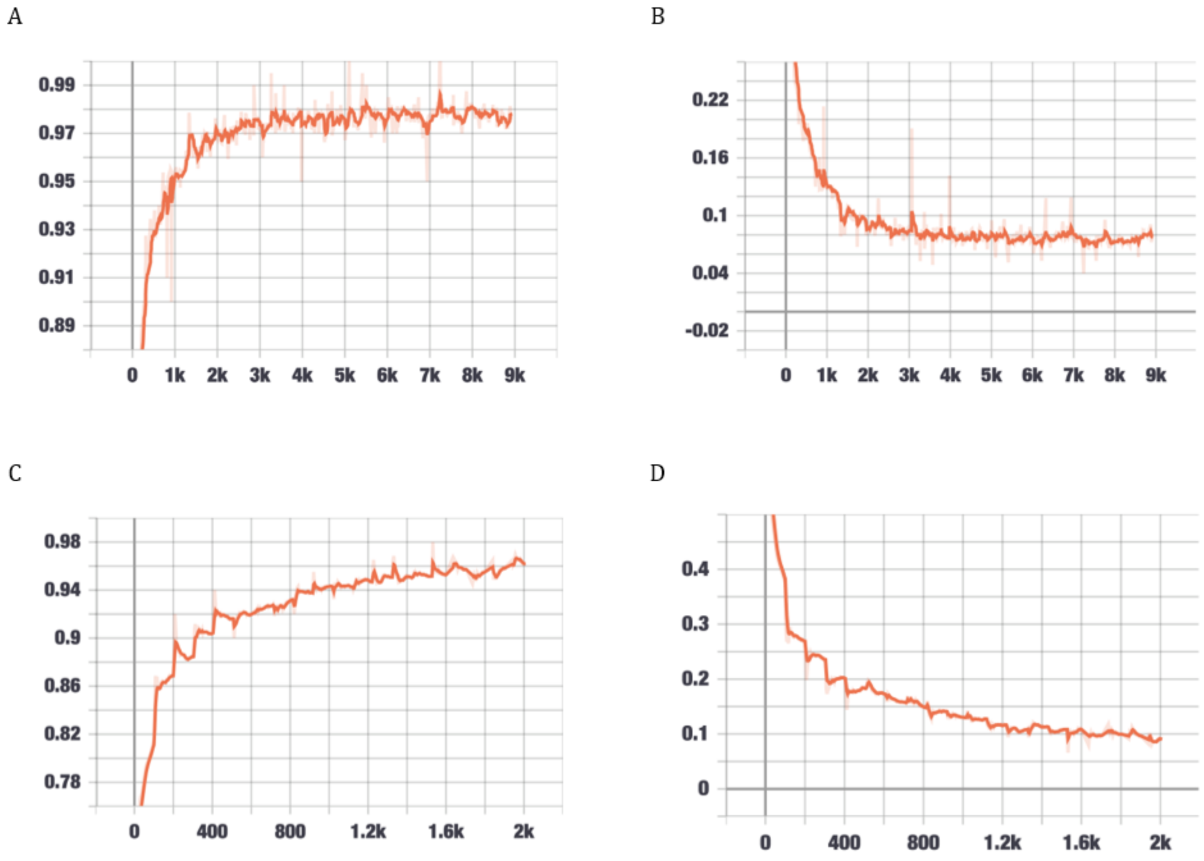

Supplementary Figure 2. Performance of HCC recognition during validation. (A) Classification accuracy is plotted against training epochs, and in (B), the categorical cross-entropy loss is shown as a function of training epochs for the binary classification problem. (C) Classification accuracy is plotted against validation epochs, and in (D), the categorical cross-entropy loss is shown as a function of validation epochs for the binary classification problem. The curve is smoothed.

2. Supplementary Tables

Supplementary Table 1. HCC image-recognition performance of the AI model and other architectures

	Sensitivity (%)	Specificity (%)	Accuracy (%)
AlexNet	97.0	95.0	96.0

GoogLeNet	98.0	96.0	97.0
Our AI Model	99.0	98.0	98.5

Supplementary Table 2. Proposed AI framework performance in classifying other types of medical images by using transfer learning

Images	Accuracy (%)	Sensitivity (%)	Specificity (%)
Colorectal cancer	96.8	97.0	96.7
Breast cancer	96.0	95.7	96.3

Supplementary Table 3. Performance of several nets at baseline

Net	Accuracy (%)
ResNet-34	98.1
ResNet-50	97.5
Inception-V4	97.9

Supplementary Table 4. Performance of several nets after tuning plus color augmentation

Net	Accuracy (%)
ResNet-34+fine-tune+color augmentation	98.5
ResNet-50+fine-tune+color augmentation	98.0
Inception-v4+fine-tune+color augmentation	98.3
